# Confidence Measurement Metrics in Multimodal Large Language Models for Ultrasound-Based Radiology Cases: Comparative Evaluation Study of Self-Reported, Consistency-Based, and Hybrid Methods

**DOI:** 10.2196/86498

**Published:** 2026-06-02

**Authors:** Taewon Han, Jaeseung Shin, Jeong Hyun Lee, Kyowon Gu

**Affiliations:** 1 Department of Radiology, Samsung Medical Center, 81 Irwon-ro, Irwon-dong - Gangnam-gu, Seoul, 06351, Republic of Korea, 82 10-8714-7650

**Keywords:** artificial intelligence, AI, radiology, medical informatics, diagnostic confidence, large language models, LLMs

## Abstract

**Background:**

Large language models (LLMs) require specialized methodologies to quantify model confidence for safe deployment in health care systems; however, there is a lack of established methods for confidence assessment.

**Objective:**

This study aimed to evaluate confidence metrics for multimodal LLMs interpreting ultrasound-based radiology cases and to compare self-reported, consistency-based, and hybrid methods.

**Methods:**

From a total of 330 quizzes on the Korean Society of Ultrasound in Medicine digital platform, we selected 94 multiple-choice cases. Four multimodal LLMs were evaluated: 3 reasoning models (GPT-5, Claude-4.5-Sonnet, and Gemini-3-Pro) and 1 general model (GPT-4o). Temperature was fixed at 1.0. Multiple confidence metrics were assessed: (1) self-reported metrics generated by LLMs using prompts that elicited direct confidence percentages with answers, including first self-reported confidence and mean self-reported confidence; (2) consistency-based metrics derived from 20 repeated outputs per case, including relative entropy calculated as 1 − H/log_2_ k (H=Shannon entropy, k=number of answer choices) and majority-vote percentage; and (3) a Top Weighted Score combining response frequency with self-reported confidence. Receiver operating characteristic analysis for discrimination and Spearman correlation between accuracy and each confidence metric was conducted. Additionally, model calibration was assessed using expected calibration error and Brier score. Processing time and token consumption (input, output, and total) were recorded for each application programming interface call to evaluate resource use across models.

**Results:**

Diagnostic accuracy varied across models, with Gemini-3-Pro achieving the highest accuracy (70/94, 74.47%), surpassing the median human accuracy (59%, IQR 40.3%-75%). Top Weighted Score, a hybrid metric combining response frequency and self-reported confidence, was the only metric achieving statistically significant correlations across all 4 models: Gemini-3-Pro (ρ=0.52), GPT-5 (ρ=0.43), Claude-4.5-Sonnet (ρ=0.30), and GPT-4o (ρ=0.22). Receiver operating characteristic analysis revealed that Top Weighted Score demonstrated the highest discriminative ability, with area under the curve values of 0.826 (95% CI 0.731‐0.920) for Gemini-3-Pro and 0.767 (95% CI 0.668‐0.866) for GPT-5. Top Weighted Score was the only metric achieving statistical significance in GPT-4o. Calibration analysis showed that Top Weighted Score achieved the lowest expected calibration error in GPT-5 (0.098) and Claude-4.5-Sonnet (0.192), while Gemini-3-Pro showed comparable calibration between relative entropy (0.119) and Top Weighted Score (0.122). Resource use analysis demonstrated that reasoning models required substantially longer processing times and higher token consumption compared to general models.

**Conclusions:**

In multimodal LLMs applied to ultrasound-based radiology cases, hybrid methods (Top Weighted Score) demonstrated significant associations across all evaluated models and appear to serve as more reliable indicators of diagnostic confidence compared to self-reported or consistency-based metrics alone, although the strength of these associations varied across models, and external validation is warranted before broader clinical application. These findings support integrative confidence estimation approaches that incorporate response consistency while highlighting the need for resource-efficient sampling strategies to enable practical clinical deployment.

## Introduction

The integration of large language models (LLMs) into clinical workflows is accelerating from promise to practice [1-3]. These advancements necessitate robust frameworks for evaluating output reliability to ensure patient safety when LLMs are used for medical decision-making [4]. Of particular concern is the calibration of LLMs—the alignment between a model’s confidence and its true accuracy—as poorly calibrated LLMs may deliver inaccurate responses with inappropriately high confidence [5], potentially introducing significant risks to patients through downstream diagnostic and treatment errors [6].

Unlike traditional probabilistic classifiers (eg, logistic regression or convolutional neural networks) that expose an explicit class probability for each prediction, LLMs generate text sequentially using probabilities but may present answers confidently despite substantial uncertainty in their underlying probability distributions, resulting in overconfidence issues [4]. This tendency toward overconfidence complicates the safe deployment of LLMs in health care systems and underscores the need for specialized methodologies to quantify model confidence for clinical end users [4].

While existing deep learning models have demonstrated established methods for uncertainty quantification in medical artificial intelligence through various approaches [7-9], architecturally different LLMs still lack standardized methodologies. Several approaches have been proposed to estimate the confidence of LLM outputs [10,11]. In the self-reported method, the model is explicitly prompted to assign a numerical confidence score, typically 0% to 100%, to its own answer [12,13]. Another approach uses sample consistency, leveraging the stochastic behavior of LLMs by running the same prompt multiple times and estimating confidence from the agreement or entropy of the resulting responses [14]. Additional methods include directly using token-level log probabilities to quantify confidence [15]. Despite these various methodologies, a best-practice standard for assessing LLM confidence has not yet been established.

Given the lack of established best practices for confidence assessment, a systematic appraisal of available techniques is essential before these systems can be deployed in clinical practice. Therefore, this study aims to evaluate confidence measurement metrics of multimodal LLMs tasked with ultrasound-based radiological cases assessing whether these approaches can serve as reliable indicators of diagnostic confidence.

## Methods

### Ethical Considerations

This investigation used publicly available educational datasets, obviating the need for institutional review board approval or informed consent. All quiz materials from the Korean Society of Ultrasound in Medicine (KSUM) website were previously deidentified before public release. No compensation was involved in this study, and no identifiable individuals appear in any images within the manuscript or supplementary materials.

### Dataset

**Figure 1.**
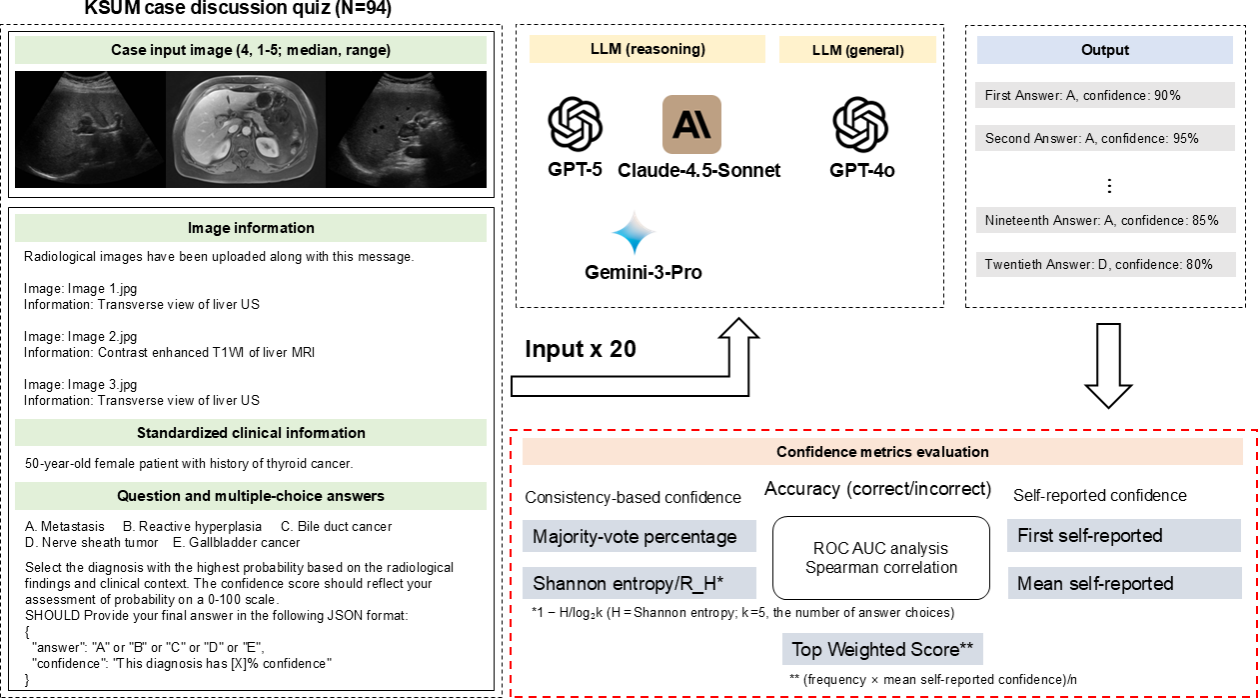
Flowchart depicting the evaluation process for consistency metrics across 3 reasoning large language models and 1 general large language model. AUC: area under the curve; KSUM: Korean Society of Ultrasound in Medicine; LLM: large language model; R_H: relative entropy; ROC: receiver operating characteristic.

A total of 330 case discussion quizzes were extracted by a radiologist (TH, with 4 years of experience) from the KSUM digital platform [16], published between July 28, 2000, and December 25, 2025. The radiologist systematically collected imaging data, question content with corresponding multiple-choice options, and relevant imaging information, including imaging modality and anatomical site. We excluded 236 cases without multiple-choice formats to maintain measurement reliability, resulting in a final dataset of 94 quiz cases (Figure 1). These quiz cases encompassed various imaging modalities, with some cases featuring challenging diagnostic scenarios. To focus on multimodal capabilities, we standardized the brief clinical text to include only patient demographics (age and sex) and chief complaint or previous medical history. The ground truth for our study was established using officially designated answers from the KSUM platform. Human performance benchmarks were derived from response statistics of KSUM platform subscribers, mainly radiologists and radiology trainees with varying degrees of expertise.

### Multimodal LLMs

We selected four multimodal LLMs, including three reasoning models—(1) GPT-5 (Alias: 2025-08-07; OpenAI) [17]; (2) Claude-4.5-Sonnet (Alias: 2025-09-29; Anthropic) [18]; (3) Gemini-3-Pro (Alias: 2025-11-18; Google) [19]—and one general model—GPT-4o (Alias: 2024-11-20; OpenAI) [20]. The temperature was fixed at 1.0 across all models because the reasoning models do not permit adjustment, and previous literature reports optimal performance at this setting [21]. Additionally, enhanced reasoning capabilities were activated for applicable models: “reasoning effort” and “thinking level” were set to “high” for GPT-5 and Gemini-3-Pro-Preview, and “thinking” mode was enabled for Claude-4.5-Sonnet.

### Confidence Measurement Metrics

Radiological images were paired with brief text prompts that described the clinical question and key imaging information. We evaluated self-reported, consistency-based, and hybrid confidence metrics while simultaneously measuring diagnostic accuracy (Figure 1). For each case, each model generated 20 independent outputs, enabling the analysis of consistency-based metrics.

First, a relative entropy–based score (R_H) was calculated as R_H=1 − H/log_₂_ k, where $H=-\sum p_{i}\log_{2}p_{i}$ represents Shannon entropy, p_*i*_ represents the relative frequency of option *i* across repeated model outputs, and k=5 is the number of answer choices (so log_₂_k≈2.322 bits). R_H ranges from 0 (maximum entropy, complete inconsistency) to 1 (zero entropy, perfect consistency); for example, a response pattern of [A A A A A] yields R_H=1, whereas [A B C D E] yields R_H=0. Intermediate patterns receive scores reflecting their coherence; [A A A B B] would yield a higher R_H value than [A A A B C] because of greater consistency. The raw Shannon entropy (H) was also reported alongside R_H to provide an unnormalized measure.

Second, a majority-vote percentage recorded the proportion of the most frequent response in repeated trials. Both [A A A B B] and [A A A B C], for instance, produce a modal proportion of 60%, illustrating that this metric ignores differences in the dispersion of the remaining responses.

Third, a weighted confidence score was calculated for each answer option as follows: (frequency × mean self-reported confidence)/n, where frequency is the count of that option across repeated trials, mean self-reported confidence is the average confidence rating for that option, and n is the total number of repetitions. The Top Weighted Score was defined as the highest score among all options. For example, if option A appeared 12 times with a mean confidence of 80% and option B appeared 8 times with a mean confidence of 90%, the weighted scores would be (12×80)/20=48 for A and (8×90)/20=36 for B, yielding a Top Weighted Score of 48.

In parallel, each model was prompted to append a numerical self-confidence rating (0%‐100%) to each answer. Two self-reported confidence metrics were derived: (1) first self-reported confidence, which used the confidence rating from the first response and (2) mean self-reported confidence, which averaged the confidence ratings across all responses that selected the majority-vote answer. The prompt instructed models to select the diagnosis based on radiological findings and clinical context before providing the final answer in JSON format, including a confidence score on a 0%‐100% scale (eg, “Select the diagnosis with the highest probability… Provide your final answer in the following JSON format: answer: A-E, confidence: 0%‐100%”). The exact templates are presented in Table S1 in Multimedia Appendix 1 and Figure 1.

To determine the minimum repetition count required for consistency-based metrics, analyses were additionally conducted with 5, 10, and 15 repeated outputs per case.

For ROC AUC, Spearman correlation, and calibration analyses, each confidence metric was paired with the diagnostic accuracy of its corresponding representative answer. For consistency-based metrics (R_H and majority-vote percentage) and mean self-reported confidence, the representative answer was the majority-voted option across repeated outputs. For the Top Weighted Score, the representative answer was the option receiving the highest weighted score. For the first self-reported confidence, the representative answer was the model’s first response. Diagnostic accuracy was assessed by comparing the corresponding representative answer for each metric with the KSUM ground truth.

### Resource Use

To evaluate the trade-off between confidence estimation reliability and resource efficiency, we recorded the processing time and token consumption for each model query. Processing time was measured as the duration from application programming interface (API) request submission to response completion. Token usage was recorded as input tokens (text prompt and image data), output tokens (model-generated response), and total tokens consumed per query. We calculated the cumulative processing time and token consumption required to analyze a single quiz case for each repetition count condition (1, 5, 10, 15, and 20).

### Statistical Analysis

Accuracy for each model and repetition count (1, 5, 10, 15, and 20) was quantified as the proportion of correct answers. Differences across repetition counts were tested using the Cochran *Q* test [22]. Discriminative ability was evaluated using receiver operating characteristic (ROC) area under the curve (AUC) with 95% CIs estimated by the DeLong method [23], using each confidence metric as a predictor of diagnostic accuracy (correct vs incorrect). Spearman correlation coefficients (ρ) measured the association between each confidence measurement and diagnostic accuracy (correct vs incorrect). Correlations were interpreted as negligible (|ρ|≤0.10), weak (0.10<|ρ|≤0.39), moderate (0.39<|ρ|≤0.69), strong (0.69<|ρ|≤0.89), or very strong (|ρ|>0.89) [24]. Calibration, defined as the alignment between predicted confidence and actual accuracy, was evaluated using expected calibration error (ECE) and Brier scores [25,26], using fixed 10-bin calibration, with 95% CIs estimated via bootstrap resampling (1000 iterations). The Brier score was calculated based on binary diagnostic accuracy (correct vs incorrect) rather than the multiclass probability across all 5 options.

Model response repeatability was evaluated using the Fleiss κ statistic, with results interpreted as follows: >0.8, almost perfect; 0.61 to 0.80, substantial; 0.41 to 0.60, moderate; 0.21 to 0.40, fair; and <0.20, poor [27]. To assess potential training data contamination, majority-vote accuracy at a repetition count of 20 was compared between cases published before February 2025 (n=72) and from February to December 2025 (n=22) using Fisher exact tests. Statistical significance was established at *P*<.05. Statistical analyses were performed using GraphPad Prism (version 10.4.1; GraphPad Software) and Python (version 3.10).

## Results

### Dataset and Diagnostic Performance

A study of 94 cases was conducted, with a median of 4 (IQR 3-4; range 1‐5) input images per case. The distribution of input image modalities comprised radiography (n=8), ultrasonography (n=94), computed tomography (n=18), magnetic resonance imaging (n=26), nuclear medicine imaging (n=6), and other diagnostic techniques, including endoscopic visualization (n=2) and aspiration fluid (n=2). Human accuracy demonstrated substantial variability (median 59%, IQR 40.3%-75%; range 5%‐96%; mean 56.8%, SD 22%).

**Figure 2.**
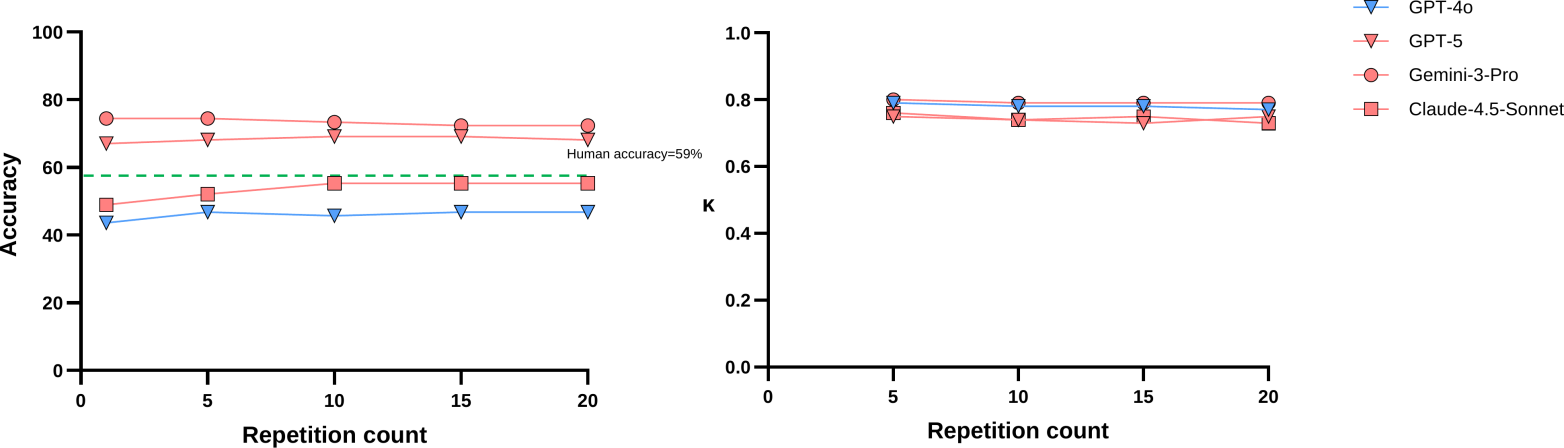
Accuracy and κ values plotted against repetition number.

Table 1 and Figure 2 demonstrate the implementation of majority voting across repetition counts. Claude-4.5-Sonnet showed a significant improvement in diagnostic accuracy with majority voting, improving from 48.94% (46/94) to 55.32% (52/94) at 10 repetitions (*P*=.01). In contrast, Gemini-3-Pro, GPT-5, and GPT-4o showed no significant change with majority voting (*P*=.67, *P*=.94, and *P*=.08, respectively), with Gemini-3-Pro showing the highest accuracy ranging from 72.34% (68/94) to 74.47% (70/94).

**Table 1.** Comparison of first output and majority vote accuracy across multimodal large language models (N=94).^a^

Model	First output, n (%)	Majority vote (5), n (%)	Majority vote (10), n (%)	Majority vote (15), n (%)	Majority vote (20), n (%)	*P* value
Claude-4.5-Sonnet	46 (48.94)	49 (52.13)	52 (55.32)	52 (55.32)	52 (55.32)	.01
Gemini-3-Pro	70 (74.47)	70 (74.47)	69 (73.40)	68 (72.34)	68 (72.34)	.67
GPT-5	63 (67.02)	64 (68.09)	65 (69.15)	65 (69.15)	64 (68.09)	.94
GPT-4o	41 (43.62)	44 (46.81)	43 (45.74)	44 (46.81)	44 (46.81)	.08

a Differences in accuracy were assessed using the Cochran *Q* test. Human accuracy for these cases showed substantial variability (median 59%, IQR 40.3%-75%; mean 56.8%, SD 22%, range 5%-96%).

Fleiss κ analysis demonstrated substantial within-model repeatability across all models (Table S2 in Multimedia Appendix 1). Gemini-3-Pro achieved the highest consistency (κ=0.79‐0.80), followed by GPT-4o (κ=0.77‐0.79). Claude-4.5-Sonnet and GPT-5 showed comparable repeatability (κ=0.73‐0.76 and κ=0.73‐0.75, respectively). All models maintained substantial agreement across all repetition counts, with response consistency remaining stable from 5 to 20 repetitions (Figure 2).

No statistically significant differences in majority-vote accuracy were observed between cases published before and after February 2025 for any of the 4 evaluated models (all *P*>.79; Table S3 in Multimedia Appendix 1).

### Confidence Measurement Metrics

The discriminative ability of confidence metrics varied substantially across models (Table 2). Top Weighted Score demonstrated the highest discriminative performance, achieving significant ROC AUC values across all models. Gemini-3-Pro showed the strongest discrimination with Top Weighted Score (ROC AUC=0.826, 95% CI 0.731‐0.920, *P*<.001), followed by GPT-5 (ROC AUC=0.767, 95% CI 0.668‐0.866, *P*<.001), Claude-4.5-Sonnet (ROC AUC=0.676, 95% CI 0.568‐0.785, *P*=.001), and GPT-4o (ROC AUC=0.629, 95% CI 0.509‐0.749, *P*=.04). Notably, Top Weighted Score was the only metric achieving statistical significance in GPT-4o.

**Table 2.** Receiver operating characteristic area under the curve comparing discriminative ability of confidence metrics in multimodal large language models.

Model	Self-reported (first) (ROC^a^ AUC^b^, 95% CI, *P* value)	Self-reported (mean) (ROC AUC, 95% CI, *P* value)	R_H^c^ (ROC AUC, 95% CI, *P* value)^d^	Majority-vote percentage (ROC AUC, 95% CI, *P* value)	Top Weighted Score (ROC AUC, 95% CI, *P* value)
Claude-4.5-Sonnet	0.706, 0.602‐0.810, <.001	0.636, 0.523‐0.748, .02	0.671, 0.565‐0.778, .002	0.668, 0.562‐0.775, .002	0.676, 0.568‐0.785, .001
Gemini-3-Pro	0.532, 0.439‐0.625, .50	0.661, 0.546‐0.775, .006	0.779, 0.672‐0.887, <.001	0.790, 0.682‐0.897, <.001	0.826, 0.731‐0.920, <.001
GPT-5	0.719, 0.613‐0.826, <.001	0.659, 0.547‐0.771, .005	0.755, 0.647‐0.863, <.001	0.740, 0.631‐0.848, <.001	0.767, 0.668‐0.866, <.001
GPT-4o	0.597, 0.491‐0.703, .07	0.592, 0.476‐0.708, .12	0.576, 0.463‐0.689, .19	0.577, 0.464‐0.689, .18	0.629, 0.509‐0.749, .04

a ROC: receiver operating characteristic.

b AUC: area under the curve.

c R_H: relative entropy.

d Shannon entropy is not reported separately because it yielded identical AUC values to the relative entropy–based score.

Consistency-based metrics (R_H, majority-vote percentage, and Shannon entropy) showed strong discriminative ability in 3 models. Gemini-3-Pro achieved the highest performance (R_H, ROC AUC=0.779, 95% CI 0.672‐0.887, *P*<.001; majority-vote percentage, ROC AUC=0.790, 95% CI 0.682‐0.897, *P*<.001; Shannon entropy, ROC AUC=0.779, 95% CI 0.672‐0.887, *P*<.001), followed by GPT-5 (R_H, ROC AUC=0.755, 95% CI 0.647‐0.863, *P*<.001; majority-vote percentage, ROC AUC=0.740, 95% CI 0.631‐0.848, *P*<.001; Shannon entropy, ROC AUC=0.755, 95% CI 0.647‐0.863, *P*<.001) and Claude-4.5-Sonnet (R_H, ROC AUC=0.671, 95% CI 0.565‐0.778, *P=*.002; majority-vote percentage, ROC AUC=0.668, 95% CI 0.562‐0.775, *P*=.002; Shannon entropy, ROC AUC=0.672, 95% CI 0.565‐0.778, *P*=.002). However, GPT-4o showed no significant discrimination with consistency-based metrics (R_H, ROC AUC=0.576, *P*=.19; majority-vote percentage, ROC AUC=0.577, *P*=.18; Shannon entropy, ROC AUC=0.576, *P*=.19).

Self-reported confidence demonstrated model-dependent performance. First self-reported confidence achieved significant discrimination in Claude-4.5-Sonnet (ROC AUC=0.706, 95% CI 0.602‐0.810, *P*<.001) and GPT-5 (ROC AUC=0.719, 95% CI 0.613‐0.826, *P*<.001). However, mean self-reported confidence showed lower discriminative ability in Claude-4.5-Sonnet (ROC AUC=0.636, *P*=.02), GPT-5 (ROC AUC=0.659, *P*=.005), and Gemini-3-Pro (ROC AUC=0.661, *P*=.006).

**Figure 3.**
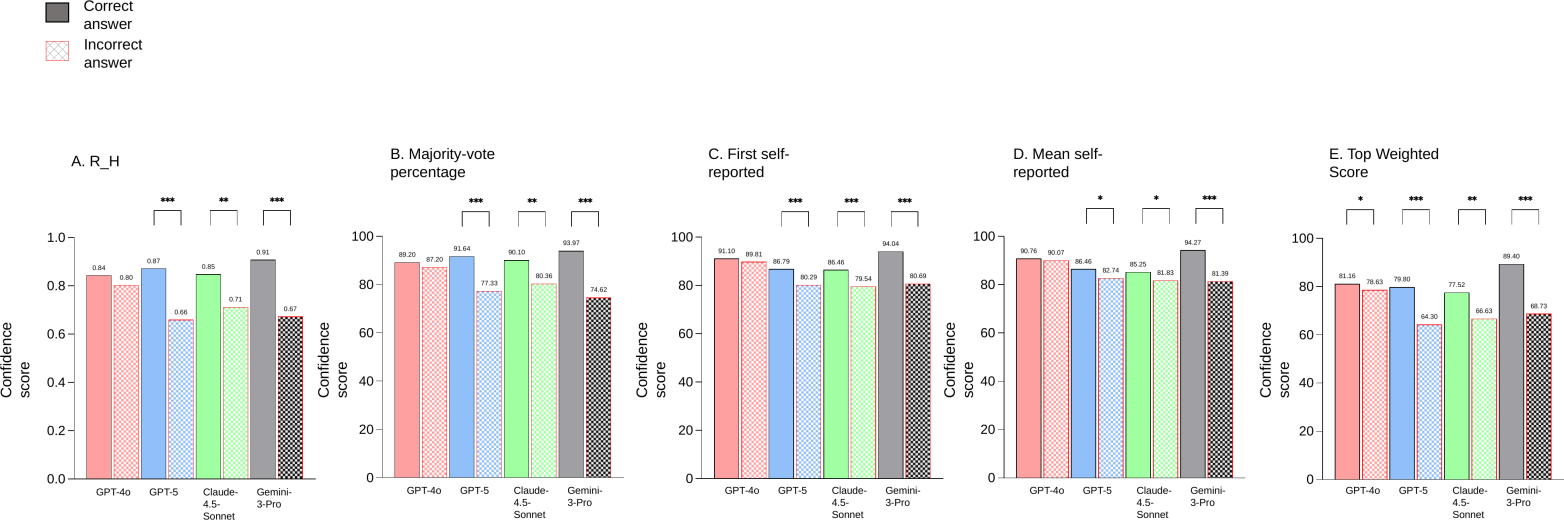
Distribution of confidence scores stratified by diagnostic accuracy (correct vs incorrect) for each multimodal large language model. (A) Relative entropy (R_H), (B) majority-vote percentage, (C) first self-reported confidence, (D) mean self-reported confidence, and (E) Top Weighted Score. Solid bars indicate correct responses; hatched bars indicate incorrect responses. Differences between groups were assessed using the Mann-Whitney *U* test. *P<.05; **P<.01; ***P<.001.

Figure 3 replicates the ROC findings. Top Weighted Score demonstrated higher confidence scores for correct responses across all 4 models, whereas R_H, majority-vote percentage, first self-reported confidence, and mean self-reported confidence showed this pattern only in GPT-5, Claude-4.5-Sonnet, and Gemini-3-Pro, excluding GPT-4o.

Spearman correlation analysis revealed similar patterns (Table 3). Notably, Top Weighted Score was the only metric achieving statistical significance across all 4 models, demonstrating moderate correlations in Gemini-3-Pro (ρ=0.52, 95% CI 0.35‐0.65, *P*<.001) and GPT-5 (ρ=0.43, 95% CI 0.25‐0.58, *P*<.001), and weak correlations in Claude-4.5-Sonnet (ρ=0.30, 95% CI 0.11‐0.48, *P*=.003) and GPT-4o (ρ=0.22, 95% CI 0.02‐0.41, *P*=.03). In GPT-4o, Top Weighted Score was the only metric achieving significance. Consistency-based metrics showed moderate correlations in Gemini-3-Pro (R_H, ρ=0.48; majority-vote percentage, ρ=0.50; all *P*<.001) and GPT-5 (R_H, ρ=0.43; majority-vote percentage, ρ=0.41; all *P*<.001). Self-reported confidence showed weak correlations across all models.

**Table 3.** Correlation analysis between accuracy and confidence metrics in multimodal large language models at a repetition count of 20.

Model and metrics	ρ (95% CI)^a^	*P* value^b^
Claude-4.5-Sonnet		
Self-reported (first)	0.36 (0.17 to 0.53)	<.001
Self-reported (mean)	0.23 (0.03 to 0.42)	.02
R_H^c^	0.31 (0.11 to 0.48)	.002
Majority-vote percentage	0.30 (0.11 to 0.48)	.003
Top Weighted Score	0.30 (0.11 to 0.48)	.003
Gemini-3-Pro		
Self-reported (first)	0.06 (−0.14 to 0.26)	.55
Self-reported (mean)	0.25 (0.05 to 0.43)	.014
R_H	0.48 (0.30 to 0.62)	<.001
Majority-vote percentage	0.50 (0.33 to 0.64)	<.001
Top Weighted Score	0.52 (0.35 to 0.65)	<.001
GPT-5		
Self-reported (first)	0.36 (0.17 to 0.52)	<.001
Self-reported (mean)	0.26 (0.06 to 0.44)	.012
R_H	0.43 (0.25 to 0.58)	<.001
Majority-vote percentage	0.41 (0.22 to 0.56)	<.001
Top Weighted Score	0.43 (0.25 to 0.58)	<.001
GPT-4o		
Self-reported (first)	0.18 (−0.02 to 0.37)	.08
Self-reported (mean)	0.16 (−0.05 to 0.35)	.13
R_H	0.14 (−0.07 to 0.33)	.18
Majority-vote percentage	0.14 (−0.06 to 0.33)	.18
Top Weighted Score	0.22 (0.02 to 0.41)	.03

a Values represent Spearman correlation coefficients (ρ) with 95% CIs in parentheses.

b Significant correlations (*P*<.05) are marked with an asterisk.

c R_H: relative entropy.

### Calibration

Calibration analysis revealed that the Top Weighted Score demonstrated the best calibration in GPT-5 (ECE=0.098, 95% CI 0.074‐0.211; Brier score=0.185, 95% CI 0.140‐0.235) and Claude-4.5-Sonnet (ECE=0.192, 95% CI 0.133‐0.307; Brier score=0.259, 95% CI 0.203‐0.317). In Gemini-3-Pro, R_H and the Top Weighted Score showed comparable calibration (ECE=0.119 vs 0.122; Brier score=0.164 vs 0.163, respectively). Across Claude-4.5-Sonnet, Gemini-3-Pro, and GPT-5, consistency-based metrics, particularly R_H, demonstrated better calibration compared to self-reported metrics. GPT-4o showed the poorest calibration across all metrics (Table 4).

**Table 4.** Calibration metrics for different confidence measurement methods in multimodal large language models at a repetition count of 20.^a^

Model and metrics	ECE^b^ (95% CI)	Brier score (95% CI)
Claude-4.5-Sonnet		
Self-reported (mean)	0.284 (0.195‐0.389)	0.317 (0.253‐0.385)
Self-reported (first)	0.340 (0.244‐0.439)	0.339 (0.275‐0.409)
R_H^c^	0.266 (0.191‐0.375)	0.288 (0.216‐0.363)
Majority-vote percentage	0.304 (0.226‐0.403)	0.321 (0.247‐0.399)
Top Weighted Score	0.192 (0.133‐0.307)	0.259 (0.203‐0.317)
Gemini-3-Pro		
Self-reported (mean)	0.216 (0.128‐0.305)	0.243 (0.165‐0.322)
Self-reported (first)	0.208 (0.120‐0.298)	0.229 (0.152‐0.313)
R_H	0.119 (0.078‐0.210)	0.164 (0.109‐0.226)
Majority-vote percentage	0.168 (0.109‐0.264)	0.178 (0.117‐0.244)
Top Weighted Score	0.122 (0.071‐0.212)	0.163 (0.109‐0.220)
GPT-5		
Self-reported (mean)	0.172 (0.097‐0.273)	0.235 (0.171‐0.303)
Self-reported (first)	0.176 (0.096‐0.278)	0.230 (0.164‐0.296)
R_H	0.140 (0.099‐0.240)	0.191 (0.135‐0.252)
Majority-vote percentage	0.206 (0.130‐0.299)	0.219 (0.154‐0.287)
Top Weighted Score	0.098 (0.074‐0.211)	0.185 (0.140‐0.235)
GPT-4o		
Self-reported (mean)	0.436 (0.329‐0.543)	0.436 (0.349‐0.523)
Self-reported (first)	0.468 (0.368‐0.570)	0.459 (0.380‐0.542)
R_H	0.377 (0.303‐0.494)	0.397 (0.313‐0.482)
Majority-vote percentage	0.413 (0.332‐0.535)	0.435 (0.345‐0.525)
Top Weighted Score	0.356 (0.272‐0.470)	0.368 (0.295‐0.440)

a Lower values indicate better calibration. ECE was calculated using 10 bins.

b ECE: expected calibration error.

c R_H: relative entropy.

### Repetition Count Analysis

**Figure 4.**
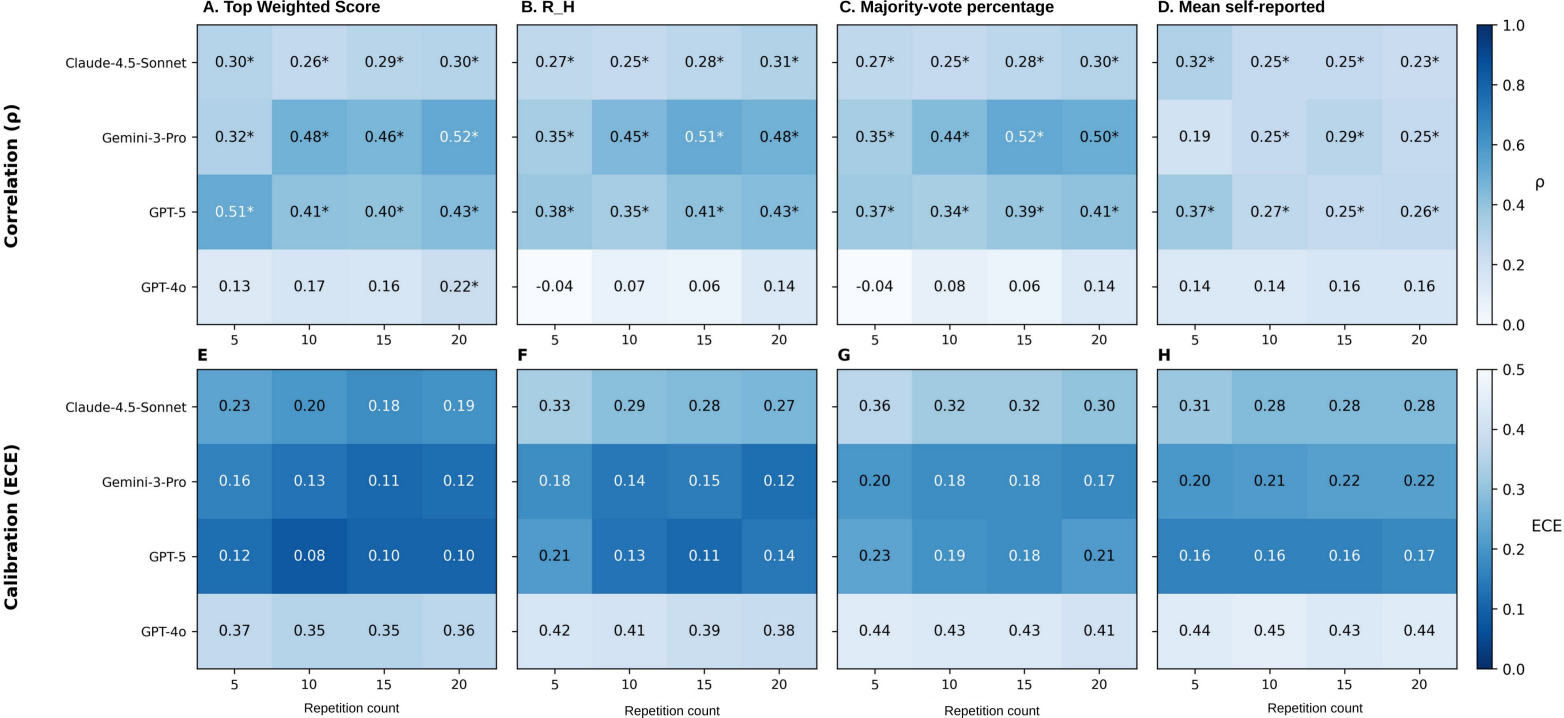
Heatmap of Spearman correlation (ρ) and calibration (ECE) between diagnostic accuracy and confidence metrics across multimodal large language models by repetition count. ECE: expected calibration error; R_H: relative entropy.

Correlation analysis stratified by repetition count revealed model-specific patterns in the relationship between consistency-based metrics and diagnostic accuracy (Table S4 in Multimedia Appendix 1). Gemini-3-Pro showed strengthening correlations with increasing repetitions, with majority-vote percentage and R_H improving from weak (ρ=0.35, *P<*.001) at 5 repetitions to moderate (ρ=0.51‐0.52, *P*<.001) at 15 repetitions, and Top Weighted Score improving from weak (ρ=0.32, *P*=.002) to moderate (ρ=0.46, *P*<.001). Mean self-reported confidence in Gemini-3-Pro achieved statistical significance only after 10 repetitions. In contrast, Claude-4.5-Sonnet demonstrated significant weak correlations across all metrics after 5 repetitions. GPT-5 showed improvement in R_H from weak (ρ=0.38, *P*<.001) at 5 repetitions to moderate (ρ=0.41, *P*<.001) at 15 repetitions, while Top Weighted Score maintained moderate correlations across all repetition counts (ρ=0.40‐0.51; all *P*<.001). GPT-4o showed nonsignificant correlations across all metrics regardless of repetition count (Figure 4).

Calibration metrics showed similar patterns with increasing repetitions (Table S5 in Multimedia Appendix 1). Top Weighted Score demonstrated decreasing ECE values across repetitions in Gemini-3-Pro (0.158 to 0.112), GPT-5 (0.118 to 0.095), and Claude-4.5-Sonnet (0.230 to 0.184).

Heatmaps display Spearman correlation coefficients (ρ; top row, panels A-D) and ECE (bottom row, panels E-H) for 4 confidence metrics across repetition counts of 5, 10, 15, and 20. For correlation, darker shading indicates a stronger positive correlation (range 0‐1); for ECE, darker shading indicates better calibration with lower error (range 0‐0.5). Statistically significant correlations (*P*<.05) are marked with an asterisk. Shannon entropy exhibited inverse correlations with identical magnitudes to R_H values (data not shown).

### Resource Use

Resource consumption varied substantially across models (Table S6 in Multimedia Appendix 1). GPT-4o demonstrated the highest efficiency, requiring a mean processing time of 6.38 (SD 2.26) seconds and a mean of 4353 (SD 1402) total tokens per case at 1 repetition, increasing to 59.43 (SD 12.46) seconds and 43,423 (SD 13,921) tokens at 10 repetitions, and 117.93 (SD 23.44) seconds and 86,877 (SD 27,869) tokens at 20 repetitions. Claude-4.5-Sonnet required a mean processing time of 29.48 (SD 6.37) seconds and a mean of 5551 (SD 1477) total tokens at 1 repetition, increasing to 288.27 (SD 44.76) seconds and 55,207 (SD 14,505) tokens at 10 repetitions, and 750.43 (SD 1694.80) seconds and 110,344 (SD 28,860) tokens at 20 repetitions. Gemini-3-Pro consumed a mean processing time of 54.75 (SD 29.79) seconds and a mean of 8774 (SD 2993) total tokens at 1 repetition, increasing to 543.87 (SD 256.35) seconds and 88,621 (SD 26,743) tokens at 10 repetitions, and 1103.97 (SD 528.83) seconds and 178,114 (SD 54,325) tokens at 20 repetitions. GPT-5 showed the highest resource consumption, requiring a mean processing time of 70.66 (SD 39.39) seconds and a mean of 6348 (SD 2026) total tokens at 1 repetition, increasing to 947.53 (SD 1808.41) seconds and 63,956 (SD 17,991) tokens at 10 repetitions, and 1768.43 (SD 2059.84) seconds and 127,879 (SD 35,210) tokens at 20 repetitions. Notably, sporadic extreme processing times exceeding 500 seconds per individual API call were observed in Claude-4.5-Sonnet (2/1880, 0.11%) calls and GPT-5 (5/1880, 0.27%) calls, whereas no such events occurred in Gemini-3-Pro or GPT-4o.

## Discussion

### Principal Findings

Our findings indicate that the Top Weighted Score, a composite metric integrating response consistency with self-reported confidence, provided the most consistent assessment of multimodal LLM output reliability for ultrasound-based radiological cases. Notably, it was the only metric to demonstrate statistically significant correlations across all 4 evaluated models, and it exhibited the best calibration in most models. In parallel, consistency-based metrics (R_H, majority-vote percentage, and Shannon entropy) showed strong discriminative performance in Gemini-3-Pro, GPT-5, and Claude-4.5-Sonnet when contrasted with self-reported confidence, underscoring the value of response agreement–derived signals for reliability estimation.

### Comparison to Prior Work

These observations align with prior studies suggesting that consistency-based calibration approaches can outperform post hoc verbalized confidence methods for estimating LLM uncertainty and reliability [11,28,29]. In radiology, Huppertz et al [30] similarly reported no significant association between verbalized confidence and diagnostic accuracy, with accuracy remaining below 50% even at the highest confidence scores. However, in our study, first-response confidence showed relatively high ROC AUC values in GPT-5 and Claude-4.5-Sonnet, whereas mean self-reported confidence demonstrated lower values; notably, Gemini-3-Pro showed the opposite pattern. This inconsistency suggests that the apparent first-response advantage may have been driven by sampling variability. Accordingly, repeated averaging of verbalized confidence scores represents an averaging of scores only partially aligned with actual correctness rather than a more accurate probability estimate [4], and this process may compress case-level variance and reduce discriminative ability. Given the intrinsically stochastic nature of LLM generation, reliance on a single initial output for confidence estimation poses significant risks in clinical settings.

Interestingly, the entropy-based metrics achieved lower ECE and Brier scores than the simple majority-vote percentage metric. This suggests that model response dispersion, rather than only the most frequent response, yields better-calibrated confidence estimates. Given the high κ values observed in our study, the benefit of entropy-based metrics is likely to increase in settings with greater response diversity, such as tasks with larger multiple-choice panels or free-text outputs. However, high interresponse agreement (κ≈0.7‐0.8) and fixed sampling parameters (eg, temperature=1) may inflate confidence and reduce discriminative power.

Additionally, although not statistically significant, Gemini-3-Pro, the highest-performing model, showed a decrease in accuracy with majority voting at 15 and 20 repetitions. This may partly reflect the systematic nature of its errors across repetitions. When interresponse agreement is high (κ=0.79‐0.80), incorrect responses tend to be consistent across repetitions, which may limit the corrective potential of majority voting and contribute to the marginal decrease in accuracy observed.

Despite broad improvements in capability across successive model releases, recent studies across domains indicate that newer models can retain a systematic tendency toward overconfidence [10,31,32]. Consistent with this literature, we observed that models frequently assigned high confidence to incorrect answers, which poses a direct challenge for deployment in clinical decision support, where confidently presented errors may be disproportionately persuasive and propagate into downstream decision-making [33,34]. Several recent studies have reported that hybrid approaches integrating consistency and verbalized confidence can outperform either method alone in certain models [4,28,35]. In our study, Top Weighted Score operationalizes a related principle by weighting candidate responses using both frequency and self-reported confidence, potentially mitigating 2 complementary limitations: (1) overconfidence inherent to verbalized estimates and (2) reduced sensitivity of pure consistency metrics when interresponse agreement is high. The consistent performance of Top Weighted Score across all tested models supports the utility of such integrative formulations for multimodal radiology tasks.

A major practical limitation of consistency-based estimation is resource intensity, as multiple independent model executions are required to compute agreement and dispersion metrics, increasing API costs and processing time [11]. In our study, processing time varied substantially by model and repetition count, with the burden particularly evident for reasoning models, where extended reasoning traces can materially increase latency and necessitate explicit cost-benefit trade-offs. To mitigate this overhead, adaptive sampling strategies have been proposed that achieve comparable accuracy with significantly reduced computational costs [35,36]. In our repetition-depth analysis, approximately 10 resampling runs per case were sufficient to stabilize discrimination and calibration estimates. Further research is needed to establish minimal sampling schedules for each model, along with inference acceleration strategies leveraging recent advances [37], to balance confidence estimation reliability with resource efficiency. Furthermore, sporadic extreme processing times were observed in Claude-4.5-Sonnet and GPT-5, likely reflecting potential API latency or timeout events. Although this processing time instability affected fewer than 0.3% of total API calls, it disproportionately inflated processing time variability and may represent a real-world barrier to clinical deployment, where predictable response times are essential.

### Limitations

This study exhibited several limitations. First, our relatively small sample size (N=94) potentially restricts the generalizability of our findings, and the fixed 10-bin ECE may be unstable in this sample, as confidence score clustering may leave some bins sparsely populated. Second, because model performance was assessed with multiple-choice questions, the evaluation does not fully represent real-world clinical scenarios that typically require free-text responses. Third, our evaluation was limited to the most widely available closed-source models and did not include open-source models that might have high potential applicability in health care. Fourth, as the KSUM educational quizzes are publicly accessible online, there is a possibility that these materials may have been included in the training corpora of the evaluated LLMs. This potential data contamination could inflate model accuracy estimates. However, a temporal holdout analysis revealed no significant accuracy differences between precutoff and postcutoff cases for any model (Table S3 in Multimedia Appendix 1). Furthermore, our previous investigation using the same dataset demonstrated no significant performance differences for GPT-4o between cases published before versus after the model’s knowledge cutoff date [38], suggesting that even if contamination occurred, its impact on model performance may be negligible given the vast scale of training parameters. Fifth, a practical limitation of consistency-based estimation is resource intensity, as multiple independent model executions are required, increasing costs and processing time. To address this, we conducted our repetition count analysis, and approximately 10 resampling runs per case appeared to be sufficient to stabilize discrimination and calibration estimates. Sixth, although processing times indicated that reasoning models engaged in extended computation, the structured JSON output requirement may have partially constrained the models’ chain-of-thought process, potentially affecting confidence calibration. Additionally, our prompt was designed such that confidence values were embedded as natural language strings within the JSON output rather than as direct numerical fields, which may introduce parsing instability if models generate slight textual variations. Seventh, our evaluation used general-purpose multimodal LLMs. Although recent studies have evaluated general-purpose multimodal LLMs on radiological cases, these investigations predominantly focus on diagnostic accuracy [3,21,38,39]. The development of radiologic domain-specific multimodal LLMs is in its early stages [40,41], and such models are not yet widely available commercially. Building upon these studies of general-purpose LLMs, our findings may contribute to the future development and evaluation of domain-specific expert models. Finally, the Top Weighted Score was developed post hoc on the study dataset, which introduces a potential risk of overfitting. Therefore, external validation using independent datasets is necessary to confirm the generalizability of this hybrid metric.

### Future Directions

Several directions for future research emerge from our findings. First, validation with larger datasets across various radiological conditions would strengthen generalizability and potentially identify additional patterns in model performance and calibration. Second, incorporating free-text tasks would better approximate real-world clinical usage. Third, comparative studies should explore whether open-source models demonstrate similar confidence calibration patterns and assess their applicability in clinical environments. Fourth, further research is needed to establish minimal sampling schedules and develop adaptive sampling strategies to balance confidence estimation reliability with resource efficiency. Additionally, studies using models with accessible internal reasoning processes could enable direct entropy computation from log probabilities, potentially reducing computational overhead while maintaining calibration accuracy. Fifth, comparative studies evaluating human reader confidence alongside LLM confidence metrics during diagnostic tasks could provide valuable insights into the potential for LLMs to augment clinical decision-making. Finally, from a clinical perspective, confidence metrics could augment human decision-making by providing reliability indicators for LLM outputs; however, clinical validation studies are needed to evaluate the practical use of these metrics in real-world diagnostic settings.

### Conclusions

In multimodal LLMs applied to ultrasound-based radiology cases, hybrid methods (Top Weighted Score) demonstrated significant associations across all evaluated models and appear to serve as more reliable indicators of diagnostic confidence compared to self-reported or consistency-based metrics alone, although the strength of this association varied across models, and external validation is warranted before broader clinical application.

## Supplementary material

10.2196/86498 Multimedia Appendix 1 Supplementary table.

10.2196/86498 Multimedia Appendix 2 Calibration new analysis code.

## Abbreviations

API: application programming interface
AUC: area under the curve
ECE: expected calibration error
KSUM: Korean Society of Ultrasound in Medicine
LLM: large language model
R_H: relative entropy
ROC: receiver operating characteristic
