# Correction: Confidence Measurement Metrics in Multimodal Large Language Models for Ultrasound-Based Radiology Cases: Comparative Evaluation Study of Self-Reported, Consistency-Based, and Hybrid Methods

**DOI:** 10.2196/104952

**Published:** 2026-07-06

**Authors:** Taewon Han, Jaeseung Shin, Jeong Hyun Lee, Kyowon Gu

**Affiliations:** 1 Department of Radiology, Samsung Medical Center, 81 Irwon-ro, Irwon-dong - Gangnam-gu, Seoul, 06351, Republic of Korea, 82 10-8714-7650

In “Confidence Measurement Metrics in Multimodal Large Language Models for Ultrasound-Based Radiology Cases: Comparative Evaluation Study of Self-Reported, Consistency-Based, and Hybrid Methods” [1], the authors noted three corrections.

First, the following footnote from Table 2 has been removed:

^c^Statistically significant results (*P*<.05) are marked with an asterisk.

Second, the Shannon entropy formula in the Confidence Measurement Metrics section currently appears as:

H=−∑ (*i*=1 to k) p*_i_* log_2_

The formula now reads:

$H=-\sum p_{i}\log_{2}p_{i}$

Last, the cell for “GPT-5” and “Majority vote (5), n (%)” in Table 1 currently reads:

64 (68)

The values in this cell have been replaced with the following:

64 (68.09)

The correction will appear in the online version of the paper on the JMIR Publications website together with the publication of this correction notice. Because this was made after submission to PubMed, PubMed Central, and other full-text repositories, the corrected article has also been resubmitted to those repositories.

## References

[1] Han T, Shin J, Lee JH, Gu K (2026). Confidence measurement metrics in multimodal large language models for ultrasound-based radiology cases: comparative evaluation study of self-reported, consistency-based, and hybrid methods. J Med Internet Res.

