# Supplementary material for: Confidence Measurement Metrics in Multimodal Large Language Models for Ultrasound-Based Radiology Cases: Comparative Evaluation Study of Self-Reported, Consistency-Based, and Hybrid Methods
Source: J Med Internet Res. 2026 Jun 2;28:e86498. doi: 10.2196/86498 (PMC13229462; doi:10.2196/86498)
Supplement: Multimedia Appendix 1 [file jmir-v28-e86498-s001.docx]

**Supplementary Table S1.** Examples of Prompts and Text Inputs for Eliciting Self-Reported Confidence

| Radiological images have been uploaded along with this message.  Image: Image 1.jpg  Information: Greyscale US axial scan at epigastric region  Image: Image 2.jpg  Information: Greyscale US sagittal scan at epigastric area  Image: Image 3.jpg  Information: Greyscale US image at intercostal view  Image: Image 4.jpg  Information: Greyscale US image at intercostal view  <image>  [Radiological images have been attached]  </image>  <clinical_context>  80-year-old female patient with abdominal discomfort.  </clinical_context>  <multiple_choice_options>  A. Lymphoma  B. Duodenal GIST  C. Gallbladder cancer with metastatic LNs  D. Pancreatic cancer with metastatic LNs  E. Xanthogranulomatous cholecystitis  </multiple_choice_options>  **Select the diagnosis with the highest probability based on the radiological findings and clinical context. The confidence score should reflect your assessment of probability on a 0-100 scale.**  **SHOULD Provide your final answer in the following JSON format:**  **{**  **"answer": "A" or "B" or "C" or "D" or "E",**  **"confidence": "This diagnosis has [X]% confidence"**  **}** |
| --- |

**Supplementary Table S2.** Response Repeatability using Fleiss’ Kappa Statistics Across Multimodal Large Language Models

| Models | Repetition 5 | Repetition 10 | Repetition 15 | Repetition 20 |
| --- | --- | --- | --- | --- |
| Claude-4.5-Sonnet | 0.76, substantial | 0.74, substantial | 0.75, substantial | 0.73, substantial |
| Gemini-3-Pro | 0.80, substantial | 0.79, substantial | 0.79, substantial | 0.79, substantial |
| GPT-5 | 0.75, substantial | 0.74, substantial | 0.73, substantial | 0.75, substantial |
| GPT-4o | 0.79, substantial | 0.78, substantial | 0.78, substantial | 0.77, substantial |

Note: The kappa coefficients were interpreted as follows: >0.80, almost perfect agreement; 0.61–0.80, substantial; 0.41–0.60, moderate; 0.21–0.40, fair; and <0.20, poor.

**Supplementary Table S3.** Comparison of Majority Vote Accuracy (at Repetition Count = 20) Between Cases Published Before February 2025 and From February to December 2025 Based on Model Knowledge Cutoff Dates

| **Model** | **Before February 2025 (n = 72)** | **February–December 2025 (n = 22)** | ***P* value** |
| --- | --- | --- | --- |
| Claude-4.5-Sonnet | 54.2% (39/72) | 59.1% (13/22) | .81 |
| Gemini-3-Pro | 72.2% (52/72) | 72.7% (16/22) | >.99 |
| GPT-5 | 66.7% (48/72) | 72.7% (16/22) | .79 |
| GPT-4o | 45.8% (33/72) | 50.0% (11/22) | .81 |

Note: *P* values were calculated using Fisher exact tests. The earliest knowledge cutoff among the four evaluated models was January 2025 (Claude-4.5-Sonnet). therefore, cases published from February 2025 onward were considered a holdout set unlikely to have been encountered during model training.

**Supplementary Table S4.** Correlation Analysis Between Accuracy and Confidence Metrics in Multimodal Large Language Models Stratified by Repetition Count

|  |  | **Repetition Count = 5** | | **Repetition Count = 10** | | **Repetition Count = 15** | | **Repetition Count = 20** | |
| --- | --- | --- | --- | --- | --- | --- | --- | --- | --- |
| **Model** | **Metrics** | **ρ (95% CI)** | ***P* value** | **ρ (95% CI)** | ***P* value** | **ρ (95% CI)** | ***P* value** | **ρ (95% CI)** | ***P* value** |
| **Claude-4.5-Sonnet** | Majority-vote percentage | 0.27  (0.07, 0.45) | .008* | 0.25  (0.05, 0.43) | .015* | 0.28  (0.08, 0.46) | .006* | 0.30  (0.11, 0.48) | .003* |
|  | R_H | 0.27  (0.07, 0.45) | .008* | 0.25  (0.05, 0.43) | .017* | 0.28  (0.08, 0.46) | .007* | 0.31  (0.11, 0.48) | .002* |
|  | Self-Reported (Mean) | 0.32  (0.12, 0.49) | .002* | 0.25  (0.05, 0.43) | .017* | 0.25  (0.05, 0.43) | .015* | 0.23  (0.03, 0.42) | .02* |
|  | Top  Weighted Score | 0.30  (0.10, 0.47) | .004* | 0.26  (0.06, 0.44) | .012* | 0.29  (0.09, 0.46) | .005* | 0.30  (0.11, 0.48) | .003* |
| **Gemini-3-Pro** | Majority-vote percentage | 0.35  (0.16, 0.52) | <.001* | 0.44  (0.27, 0.59) | <.001* | 0.52  (0.36, 0.65) | <.001* | 0.50  (0.33, 0.64) | <.001* |
|  | R_H | 0.35  (0.16, 0.52) | <.001* | 0.45  (0.27, 0.59) | <.001* | 0.51  (0.34, 0.64) | <.001* | 0.48  (0.30, 0.62) | <.001* |
|  | Self-Reported (Mean) | 0.19  (-0.01, 0.38) | .06 | 0.25  (0.05, 0.43) | .015* | 0.29  (0.09, 0.46) | .005* | 0.25  (0.05, 0.43) | .014* |
|  | Top  Weighted Score | 0.32  (0.13, 0.49) | .002* | 0.48  (0.31, 0.62) | <.001* | 0.46  (0.29, 0.61) | <.001* | 0.52  (0.35, 0.65) | <.001* |
| **GPT-5** | Majority-vote percentage | 0.37  (0.19, 0.54) | <.001* | 0.34  (0.15, 0.51) | <.001* | 0.39  (0.20, 0.55) | <.001* | 0.41  (0.22, 0.56) | <.001* |
|  | R_H | 0.38  (0.19, 0.54) | <.001* | 0.35  (0.16, 0.52) | <.001* | 0.41  (0.23, 0.57) | <.001* | 0.43  (0.25, 0.58) | <.001* |
|  | Self-Reported (Mean) | 0.37  (0.18, 0.53) | <.001* | 0.27  (0.06, 0.44) | .013* | 0.25  (0.05, 0.43) | .016* | 0.26  (0.06, 0.44) | .012* |
|  | Top  Weighted Score | 0.51  (0.34, 0.65) | <.001* | 0.41  (0.23, 0.57) | <.001* | 0.40  (0.21, 0.56) | <.001* | 0.43  (0.25, 0.58) | <.001* |
| **GPT-4o** | Majority-vote percentage | -0.04  (-0.24, 0.17) | .71 | 0.08  (-0.13, 0.27) | .48 | 0.06  (-0.14, 0.26) | .55 | 0.14  (-0.06, 0.33) | .18 |
|  | R_H | -0.04  (-0.24, 0.16) | .69 | 0.07  (-0.14, 0.26) | .54 | 0.06  (-0.15, 0.26) | .57 | 0.14  (-0.07, 0.33) | .18 |
|  | Self-Reported (Mean) | 0.14  (-0.07, 0.33) | .19 | 0.14  (-0.07, 0.33) | .19 | 0.16  (-0.05, 0.35) | .13 | 0.16  (-0.05, 0.35) | .13 |
|  | Top  Weighted Score | 0.13  (-0.08, 0.32) | .23 | 0.17  (-0.02, 0.37) | .07 | 0.16  (-0.05, 0.35) | .13 | 0.22  (0.02, 0.41) | .03* |

*Statistically significant correlation (*P*<.05).

Note: All values represent Spearman's rank correlation coefficients (ρ) with 95% confidence intervals. Analyses were performed between diagnostic accuracy and confidence metrics across repetition count conditions.

**Supplementary Table S5.** Calibration Metrics of Confidence Measurements in Multimodal Large Language Models Stratified by Repetition Count

|  |  | **Repetition Count**  **= 5** | | **Repetition Count**  **= 10** | | **Repetition Count**  **= 15** | | **Repetition Count**  **= 20** | |
| --- | --- | --- | --- | --- | --- | --- | --- | --- | --- |
| **Model** | **Parameter** | **ECE**  **(95% CI)** | **Brier**  **(95% CI)** | **ECE**  **(95% CI)** | **Brier**  **(95% CI)** | **ECE**  **(95% CI)** | **Brier**  **(95% CI)** | **ECE**  **(95% CI)** | **Brier**  **(95% CI)** |
| **Claude-4.5-Sonnet** | Majority-vote percentage | 0.364 (0.274, 0.466) | 0.365 (0.285, 0.452) | 0.317 (0.241, 0.421) | 0.338 (0.264, 0.422) | 0.318 (0.243, 0.420) | 0.330 (0.256, 0.411) | 0.304 (0.226, 0.403) | 0.321 (0.247, 0.399) |
|  | R_H | 0.331 (0.247, 0.436) | 0.346 (0.266, 0.431) | 0.294 (0.209, 0.395) | 0.312 (0.240, 0.390) | 0.281 (0.209, 0.390) | 0.302 (0.228, 0.377) | 0.266 (0.191, 0.375) | 0.288 (0.216, 0.363) |
|  | Self-Reported (Mean) | 0.312 (0.218, 0.411) | 0.329 (0.264, 0.397) | 0.283 (0.195, 0.382) | 0.316 (0.252, 0.384) | 0.285 (0.198, 0.389) | 0.316 (0.252, 0.383) | 0.284 (0.195, 0.389) | 0.317 (0.253, 0.385) |
|  | Top  Weighted Score | 0.230 (0.153, 0.336) | 0.279 (0.221, 0.337) | 0.195 (0.141, 0.315) | 0.268 (0.213, 0.328) | 0.184 (0.120, 0.290) | 0.262 (0.206, 0.320) | 0.192 (0.133, 0.307) | 0.259 (0.203, 0.317) |
| **Gemini-3-Pro** | Majority-vote percentage | 0.196 (0.126, 0.287) | 0.203 (0.138, 0.276) | 0.177 (0.112, 0.263) | 0.190 (0.128, 0.257) | 0.182 (0.125, 0.274) | 0.176 (0.115, 0.244) | 0.168 (0.109, 0.264) | 0.178 (0.117, 0.244) |
|  | R_H | 0.182 (0.113, 0.269) | 0.190 (0.129, 0.257) | 0.141 (0.087, 0.233) | 0.176 (0.118, 0.239) | 0.147 (0.097, 0.234) | 0.167 (0.111, 0.230) | 0.119 (0.078, 0.210) | 0.164 (0.109, 0.226) |
|  | Self-Reported (Mean) | 0.196 (0.112, 0.282) | 0.225 (0.151, 0.301) | 0.206 (0.121, 0.297) | 0.233 (0.158, 0.312) | 0.216 (0.128, 0.305) | 0.242 (0.164, 0.321) | 0.216 (0.128, 0.305) | 0.243 (0.165, 0.322) |
|  | Top  Weighted Score | 0.158 (0.092, 0.249) | 0.188 (0.129, 0.252) | 0.129 (0.078, 0.226) | 0.172 (0.116, 0.231) | 0.112 (0.070, 0.207) | 0.164 (0.110, 0.224) | 0.122 (0.071, 0.212) | 0.163 (0.109, 0.220) |
| **GPT-5** | Majority-vote percentage | 0.228 (0.147, 0.323) | 0.235 (0.165, 0.311) | 0.193 (0.120, 0.287) | 0.216 (0.151, 0.291) | 0.177 (0.112, 0.275) | 0.212 (0.149, 0.279) | 0.206 (0.130, 0.299) | 0.219 (0.154, 0.287) |
|  | R_H | 0.210 (0.133, 0.306) | 0.223 (0.157, 0.295) | 0.128 (0.096, 0.245) | 0.200 (0.141, 0.266) | 0.111 (0.088, 0.230) | 0.186 (0.131, 0.243) | 0.140 (0.099, 0.240) | 0.191 (0.135, 0.252) |
|  | Self-Reported (Mean) | 0.164 (0.092, 0.267) | 0.224 (0.165, 0.290) | 0.162 (0.083, 0.258) | 0.227 (0.166, 0.292) | 0.160 (0.085, 0.258) | 0.227 (0.166, 0.292) | 0.172 (0.097, 0.273) | 0.235 (0.171, 0.303) |
|  | Top  Weighted Score | 0.118 (0.080, 0.215) | 0.181 (0.133, 0.233) | 0.084 (0.064, 0.193) | 0.184 (0.139, 0.235) | 0.095 (0.071, 0.203) | 0.183 (0.140, 0.231) | 0.098 (0.074, 0.211) | 0.185 (0.140, 0.235) |
| **GPT-4o** | Majority-vote percentage | 0.436 (0.328, 0.547) | 0.462 (0.364, 0.558) | 0.435 (0.343, 0.551) | 0.450 (0.358, 0.543) | 0.431 (0.348, 0.555) | 0.444 (0.352, 0.536) | 0.413 (0.332, 0.535) | 0.435 (0.345, 0.525) |
|  | R_H | 0.423 (0.321, 0.538) | 0.457 (0.363, 0.551) | 0.405 (0.307, 0.526) | 0.432 (0.343, 0.523) | 0.391 (0.305, 0.510) | 0.416 (0.331, 0.504) | 0.377 (0.303, 0.494) | 0.397 (0.313, 0.482) |
|  | Self-Reported (Mean) | 0.436 (0.329, 0.543) | 0.436 (0.351, 0.523) | 0.446 (0.342, 0.552) | 0.445 (0.362, 0.529) | 0.435 (0.329, 0.542) | 0.436 (0.349, 0.523) | 0.436 (0.329, 0.543) | 0.436 (0.349, 0.523) |
|  | Top  Weighted Score | 0.369 (0.276, 0.477) | 0.389 (0.313, 0.463) | 0.349 (0.267, 0.474) | 0.380 (0.304, 0.455) | 0.347 (0.274, 0.469) | 0.376 (0.303, 0.449) | 0.356 (0.272, 0.470) | 0.368 (0.295, 0.440) |

Note: ECE = Expected Calibration Error; Brier = Brier Score. Lower values indicate better calibration.

**Supplementary Table S6. Resource Usage Across Multimodal Large Language Models Stratified by Repetition Count**

| **Model** | **Repetition Count** | **Processing Time (sec)** | **Input Tokens** | **Output Tokens** | **Total Tokens** |
| --- | --- | --- | --- | --- | --- |
| **Claude-4.5-Sonnet** | 1 | 29.48 ± 6.37 | 4,100 ± 1,393 | 1,451 ± 303 | 5,551 ± 1,477 |
|  | 5 | 144.81 ± 23.59 | 20,500 ± 6,966 | 7,129 ± 1,117 | 27,629 ± 7,239 |
|  | 10 | 288.27 ± 44.76 | 41,000 ± 13,931 | 14,207 ± 2,129 | 55,207 ± 14,505 |
|  | 15 | 607.39 ± 1,695.17 | 61,500 ± 20,897 | 21,292 ± 3,211 | 82,792 ± 21,694 |
|  | 20 | 750.43 ± 1,694.80 | 82,000 ± 27,862 | 28,344 ± 4,156 | 110,344 ± 28,860 |
| **Gemini-3-Pro*** | 1 | 54.75 ± 29.79 | 4,138 ± 1,258 | 658 ± 99 | 8,774 ± 2,993 |
|  | 5 | 271.10 ± 127.96 | 20,691 ± 6,291 | 3,263 ± 376 | 44,164 ± 13,350 |
|  | 10 | 543.87 ± 256.35 | 41,382 ± 12,582 | 6,510 ± 743 | 88,621 ± 26,743 |
|  | 15 | 824.54 ± 400.67 | 62,073 ± 18,873 | 9,763 ± 1,108 | 133,261 ± 40,658 |
|  | 20 | 1,103.97 ± 528.83 | 82,764 ± 25,164 | 13,010 ± 1,476 | 178,114 ± 54,325 |
| **GPT-5** | 1 | 70.66 ± 39.39 | 3,392 ± 1,144 | 2,956 ± 1,547 | 6,348 ± 2,026 |
|  | 5 | 590.12 ± 1,794.32 | 16,959 ± 5,721 | 15,144 ± 6,697 | 32,103 ± 9,350 |
|  | 10 | 947.53 ± 1,808.41 | 33,919 ± 11,441 | 30,037 ± 12,413 | 63,956 ± 17,991 |
|  | 15 | 1,308.79 ± 1,834.90 | 50,878 ± 17,162 | 45,196 ± 18,509 | 96,074 ± 26,666 |
|  | 20 | 1,768.43 ± 2,059.84 | 67,837 ± 22,883 | 60,042 ± 24,273 | 127,879 ± 35,210 |
| **GPT-4o** | 1 | 6.38 ± 2.26 | 4,064 ± 1,385 | 289 ± 98 | 4,353 ± 1,402 |
|  | 5 | 30.00 ± 6.98 | 20,319 ± 6,927 | 1,416 ± 303 | 21,735 ± 6,986 |
|  | 10 | 59.43 ± 12.46 | 40,639 ± 13,853 | 2,785 ± 548 | 43,423 ± 13,921 |
|  | 15 | 88.89 ± 17.86 | 60,958 ± 20,780 | 4,186 ± 787 | 65,144 ± 20,888 |
|  | 20 | 117.93 ± 23.44 | 81,277 ± 27,707 | 5,600 ± 1,027 | 86,877 ± 27,869 |

**Note:** Values represent the cumulative resources required to analyze a single quiz case at each repetition count—calculated as the sum of processing time and tokens across iterations 1 to N, averaged over 94 quiz cases. Values are presented as mean ± standard deviation across 94 quiz cases. *For Gemini-3-Pro, the total token count includes internal reasoning tokens (thinking tokens) that are not reflected in the input or output token columns.
